# Associations between burnout and career disengagement factors among general practitioners: a path analysis

**DOI:** 10.3389/fpubh.2025.1547102

**Published:** 2025-06-18

**Authors:** Christos Grigoroglou, Mark Hann, Alexander Hodkinson, Salwa S. Zghebi, Evangelos Kontopantelis, Darren M. Ashcroft, Carolyn A. Chew-Graham, Rupert A. Payne, Paul Little, Simon de Lusignan, Anli Yue Zhou, Aneez Esmail, Maria Panagioti

**Affiliations:** ^1^Division of Population Health, Health Services Research and Primary Care, National Institute for Health and Care Research (NIHR) School for Primary Care Research, University of Manchester, Manchester, United Kingdom; ^2^Division of Population Health, Health Services Research and Primary Care, Manchester Centre for Health Economics, University of Manchester, Manchester, United Kingdom; ^3^Division of Population Health, Health Services Research and Primary Care, Centre for Biostatistics, University of Manchester, Manchester, United Kingdom; ^4^Division of Pharmacy and Optometry, National Institute for Health and Care Research Greater Manchester Patient Safety Research Collaboration, University of Manchester, Manchester, United Kingdom; ^5^Division of Informatics, Imaging and Data Sciences, University of Manchester, Manchester, United Kingdom; ^6^Centre for Pharmacoepidemiology and Drug Safety, School of Health Sciences, Faculty of Biology, Medicine and Health, University of Manchester, Manchester, United Kingdom; ^7^School of Medicine, Keele University, Keele, United Kingdom; ^8^Exeter Collaboration for Academic Primary Care, University of Exeter, Exeter, United Kingdom; ^9^Primary Care Research Centre, University of Southampton, Southampton, United Kingdom; ^10^Nuffield Department of Primary Care Health Sciences, University of Oxford, Oxford, United Kingdom; ^11^Royal College of General Practitioners Research and Surveillance Centre, London, United Kingdom

**Keywords:** burnout, general practice, workforce, structural equation modelling, well-being

## Abstract

**Background:**

Burnout is associated with career disengagement among general practitioners (GPs), but the underlying mechanisms of this association remain poorly understood.

**Objective:**

This study examined the pathways linking burnout to career disengagement factors among GPs.

**Methods:**

An 11-item online questionnaire, including validated abbreviated measures of burnout outcomes (single items on emotional exhaustion (EE) and depersonalisation), career disengagement factors (intention to quit patient care, work–life balance, presenteeism and job satisfaction), and demographic information, was distributed to a random sample of GPs in England between December 2019 and April 2020. Correlations between burnout outcomes and disengagement factors were assessed, followed by a path analysis using a generalized structural equation model, to examine directional relationships between burnout outcomes and survey variables.

**Results:**

A total of 351 GPs from 57 different medical practices completed the questionnaire. Up to one in four GPs (22.5%) experienced emotional exhaustion, while up to one in three (27.4%) experienced depersonalisation on a weekly basis. In addition, one in three GPs (33.3%) expressed a moderate-to-high intention to quit patient care within the next 5 years. Moreover, one in five GPs (18.8%) reported job dissatisfaction, two in five GPs (40.7%) indicated poor work–life balance, and up to one in two GPs (27.4%) reported presenteeism in the past year. In the path analysis, intention to quit patient care had significant direct associations with both job satisfaction and burnout and significant indirect associations (via burnout) with work–life balance and presenteeism. GP demographics were excluded from the path analysis because they exhibited very weak correlations with dimensions of burnout and work engagement factors.

**Conclusion:**

These findings highlight the urgent need for interventions and policies aimed at addressing burnout and improving job satisfaction to retain GPs. In addition, improving work–life balance and reducing presenteeism could serve as effective early preventative measures to reduce burnout and job dissatisfaction and, in turn, retain GPs.

## Introduction

There is substantial evidence, both internationally and within the United Kingdom (UK), indicating a workforce crisis in primary care (1, 2). More than half of general practitioners (GPs) have reported experiencing burnout symptoms, which has led many to disengage from practice by opting for part-time work, considering early retirement, or intending to quit medical practice (3–5). The Job Demands-Resources model offers a useful framework to understand how the imbalance between job demands (such as workload, emotional strain and poor work–life balance) and available resources (including job satisfaction) contributes to burnout and subsequent career disengagement (6). Common indicators of potential career disengagement, as outlined in recent frameworks (7), include low job satisfaction, presenteeism/absenteeism, poor work–life balance, and intention to quit patient care. However, intention to quit patient care was a strong indicator of actual turnover rates among healthcare professionals, including GPs (8, 9).

Research suggests that emotional exhaustion (EE), a dimension of burnout, is strongly associated with these career disengagement factors (5). International studies have demonstrated robust associations between burnout and career disengagement factors, such as job dissatisfaction, poor work–life balance, presenteeism, and turnover intention among healthcare workers (7, 10–13). For instance, low job satisfaction correlates with burnout in physicians, and those experiencing high burnout levels are more inclined to express the intention to quit patient care (10, 13, 14). However, formal path analyses that examine the strength and direction of these associations are scarce in the literature (15), particularly among GPs in UK general practices. Understanding these pathways is crucial for guiding policymakers in identifying the association between dimensions of burnout, career disengagement factors, and GP/practice factors that require urgent attention to improve GP retention rates (16).

In this study, a custom questionnaire was distributed to a random sample of GPs in England to investigate, through path analysis, the association between burnout (measured by emotional exhaustion and depersonalisation), demographic factors (age distribution, gender composition, time commitment, and years in practice), and career disengagement factors (including work–life balance, intention to quit patient care, presenteeism, and job satisfaction) among GPs.

## Methods

### Data collection

#### Study design and sample

This cross-sectional study was conducted in England based on responses from a GP questionnaire. The Royal College of General Practitioners (RCGP) Research and Surveillance Centre (RSC), which manages data collection and monitoring from over 2,000 general practices across England and Wales, facilitated the recruitment process for the research team between December 2019 and April 2020. The RCGP RSC sent invitation letters to practice managers at all practices in its network, inviting them to participate in an online survey hosted on the SurveyMonkey platform (17, 18).

The first 70 practices that volunteered were included in the study. The research team aimed to recruit approximately 350–400 GPs from these practices. The target number of GPs was determined based on available funding rather than a formal sample size calculation. Within each participating practice, deterministic sampling was employed in-house to mitigate potential selection bias. This approach assumes a random distribution based on discrete weighted samples and helps predict statistical moments that represent the properties of the overall distribution.

### Questionnaire

The research team developed a cross-sectional questionnaire to examine burnout and career disengagement factors among GPs in England. The questionnaire consisted entirely of previously validated items (19–22).

The questionnaire comprised 11 items, including the following:

- A validated abbreviated scale from the Maslach Burnout Inventory (MBI), consisting of two items—emotional exhaustion and depersonalisation—was used. These items were measured on a 7-point ordinal scale (from never to every day), with higher scores indicating greater burnout. This abbreviated measure has been validated in many studies involving GPs and other clinicians (11, 23, 24). These two single items provide more meaningful insights into burnout among medical professionals compared to the full MBI (19). In addition, several studies have demonstrated that these two dimensions have the highest factor loadings in terms of their respective burnout domains (23, 25).
- Validated questions on intention to quit direct patient care, job satisfaction, work–life balance, and presenteeism (four items) were included. These items were sourced from previously published instruments that have been validated for their association with burnout (19–22).
- GP and practice characteristics such as age, gender, full-time equivalent (FTE), sum of FTE of all GPs in the practice and years worked in the practice (five items), were included.

The scales and items underwent discussion and agreed upon through consensus within the research group through patient and public involvement (PPI) and stakeholder engagement with GPs. The questionnaire is provided in Supplementary information S1. Completion of the questionnaire required 3–4 min. Participation in the questionnaire was voluntary, and written informed consent was obtained from all participants on the first page of the questionnaire. In December 2019, all general practice managers with valid email addresses received an email containing a link to the electronically administered questionnaire, which they were asked to circulate to GPs in their practices. All GPs, regardless of employment status (full-time, part-time, salaried, partners, or locums), were eligible to participate in the study. Non-respondents received reminders at 2-week and 4-week intervals, with data collection concluding in April 2020. The questionnaire link was personalized with a unique serial number but no personal identifiers. Participants who completed the questionnaire received a £20 payment reimbursement.

### Statistical analyses

First, we summarized the age distribution, gender composition, time commitment, and years in practice of the respondents, along with the distribution of responses to key survey variables. Pairwise correlations between the two burnout dimensions and career disengagement factors were calculated using Kendall’s tau-b due to the ordinal nature of the data. To determine the presence of within-practice clustering in the responses to the survey questions, we calculated the intra-class correlation using a one-way ANOVA, which was calculated separately for all practices and for practices with at least two, three, five and seven responses. We used a multi-level ordered logistic regression to assess whether GP demographics had a discernible effect on career disengagement factors.

**Figure 1:**
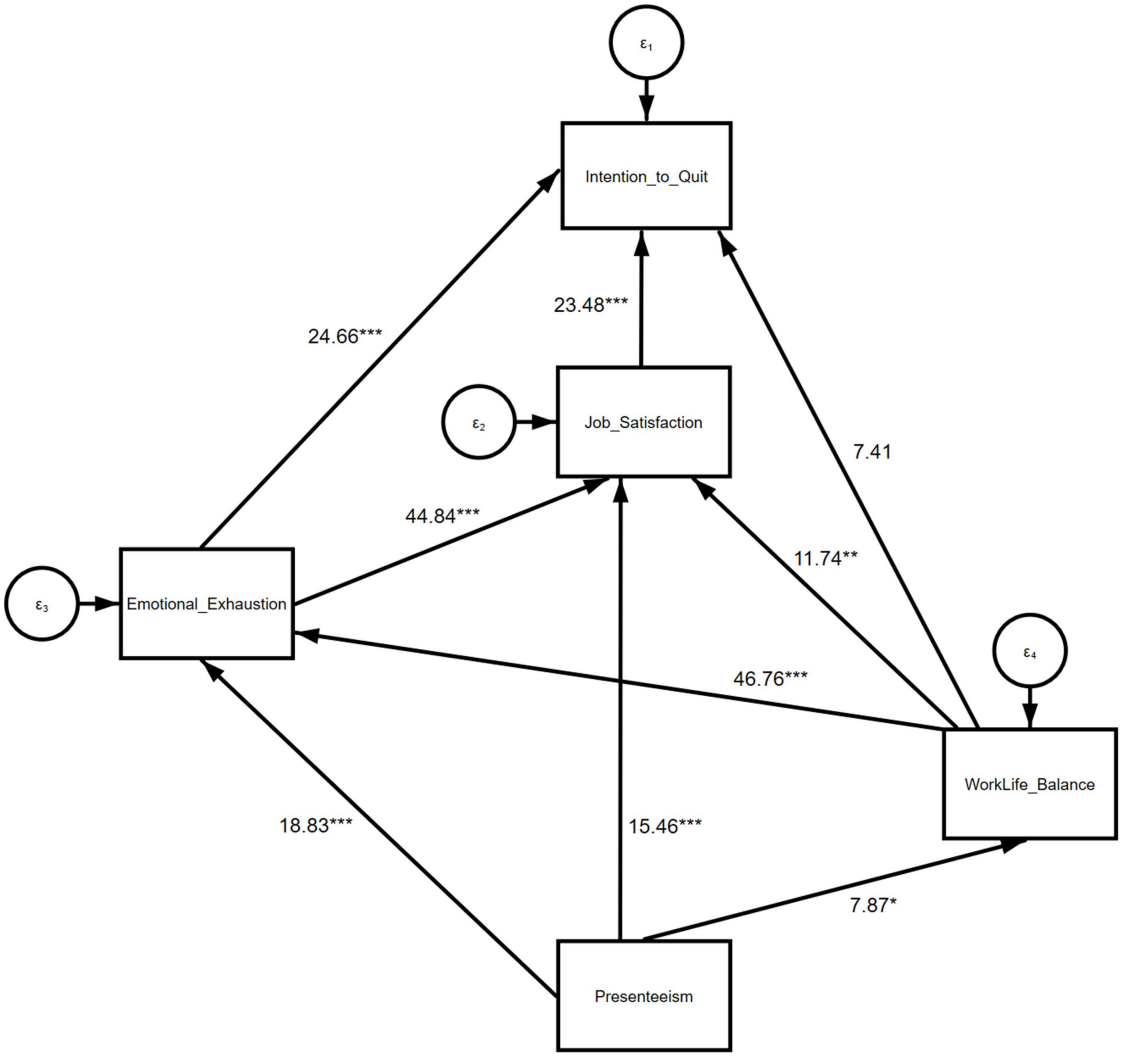
Structural model with emotional exhaustion frequency as the outcome^†^. ^†^*n* = 351; values represent the chi-squared test for statistical significance, where **p ≤ 0.001, **p < 0.01, *p < 0.05.

Next, we employed generalized structural equation modelling (GSEM) in Stata to examine pre-specified directional relationships between the survey variables, as depicted in the path diagram in Figure 1. Two models examining different burnout dimensions were utilized: one investigating the relationship between emotional exhaustion frequency and career disengagement factors and another exploring the relationship between depersonalisation frequency and career disengagement factors. As some of the variable scores were missing from the original survey, missing data were imputed using the R package ‘MICE: Multivariate Imputation by Chained Equations’ (26).

GSEM enables the treatment of variables as ordinal within an ordered logistic regression; however, this approach entails estimating a large number of parameters. Model comparisons were based on log-likelihoods and available degrees of freedom, as conventional model-fit statistics are not applicable to GSEM. To streamline the model and prevent potential issues with convergence or estimation imprecision, GP demographic variables were omitted, as their inclusion could overly complicate the model (27). Due to the large number of estimated parameters, we performed chi-squared tests to assess the statistical significance of the associations between the response items and used *p*-values to identify the strongest associations of interest.

Furthermore, we considered models for the subset of GPs aged 50 years or younger, as this group was presumed to be less likely to express a desire to quit general practice due to retirement, potentially making them more inclined to report wanting to quit due to job-related factors. This approach allowed us to better focus on GPs whose intention to quit patient care were more likely to be related to job factors, such as burnout or work dissatisfaction.

### Results

A total of 351 GPs from 57 general practices completed the bespoke questionnaire. The questionnaire was distributed to 67 practices, but 10 (15%) practices were excluded as only one GP registrar responded from these practices, and they were consulting at multiple locations, making it impossible to assign them a unique practice ID. The median response rate of the GPs across the 57 practices was 39% (range 12–91%). The average age of the participants was 45 years (SD = 8.5, range = 28 to 70), with 56% (196 out of 351) being female. Regarding work experience, 7.4% (26 GPs) had been in practice for less than 1 year, 73% (256 GPs) for 1 to 20 years, and 19.7% (69 GPs) for more than 20 years. Data analysis was based on 351 responses, except for the analysis that involved the intention to quit, which included 348 responses. The results are presented in Table 1, which indicated that between one in four (25%) and one in five (20%) GP participants reported feeling emotionally exhausted on a weekly basis, while between one in three (33%) and one in four (25%) GPs reported feeling callous toward other people at least once a week. Regarding career disengagement factors, one in three (33%) participants indicated having at least a moderate intention to quit direct patient care in the next 5 years, and one in five (20%) participants reported dissatisfaction with their career in general practice. In terms of work–life balance, two in five (40%) respondents indicated that their work schedule did not afford them enough time for personal/family life, and between one in two (50%) and one in three (33%) reported working while ill (presenteeism) at least 2–5 times over the last 12 months. We observed weak to moderate correlations between the key variables of interest (Supplementary Table S2) and very low intra-class correlation coefficients for the items in all four categories (i.e., all practices and practices with more than two, three, five or seven employed GPs) (Supplementary Table S3). The results from the multi-level ordered logistic regression exploring the effects of GP demographics on career disengagement factors indicated no statistically significant associations, as presented in Supplementary Table S1.

**Table 1:** Descriptive statistics.*

Frequency	*N*	%
Emotional exhaustion FREQ^ⱡ^		
Never	34	9.7
Less than a few times a year	108	30.8
Less than once a month	58	16.5
A few times a month	72	20.5
Once a week	32	9.1
A few times a week	38	10.8
Every day	9	2.6
ⱡweekly or more frequently = 22.5% (between one in four and one in five respondents)		
Depersonalisation^±^		
Never	70	19.9
Less than a few times a year	112	31.9
Less than once a month	60	17.1
A few times a month	13	3.7
Once a week	54	15.4
A few times a week	32	9.7
Every day	8	2.3
ⱡweekly or more frequently = 27.4% (between 1 in 3 and 1 in 4 respondents)		
Intention to quit^†^		
None	116	33.3
Slight	116	33.3
Moderate	49	14.1
Considerable	34	9.8
High	33	9.5
†moderate, considerable, or high = 33.4% (one in three respondents)		
Job satisfaction^¥^		
Strongly agree	49	14.0
Agree	162	46.2
‘Neutral’	74	21.1
Disagree	52	14.8
Strongly disagree	14	4.0
¥ (strongly) disagree = 18.8% (1 in 5 respondents)		
Work–life balance^††^		
Strongly agree	27	7.7
Agree	107	30.5
‘Neutral’	74	21.1
Disagree	97	27.6
Strongly disagree	46	13.1
†† (strongly) disagree = 40.7% (2 in 5 respondents)		
Presenteeism		
Never	94	26.8
Once	113	32.2
2 to 5 times	122	34.8
More than 5 times	22	6.3
ⱡ2 to 5 times or more frequently = 27.4% (between one in two and 1 in 3 respondents)		
Age *(mean, sd)*	Mean = 45, sd = 8.5	
Female *(%)*	56% (N = 197)	
Years in practice *(N)*	< 1 year = 26 GPs, 1 to 20 years = 256 GPs, >20 years = 69 GPs	
FTE *(% of all responses)*	FTE ≤ 50%: 23.7%; 50% < FTE ≤ 75%: 34.3%; FTE > 75%: 42%	

*All percentages are based on *N* = 351, except for ITQ, which is based on *N* = 348 (percentages may not sum to 100 due to rounding ‘errors’).

### Path relationship between job satisfaction, burnout, and other career disengagement factors

**Figure 2:**
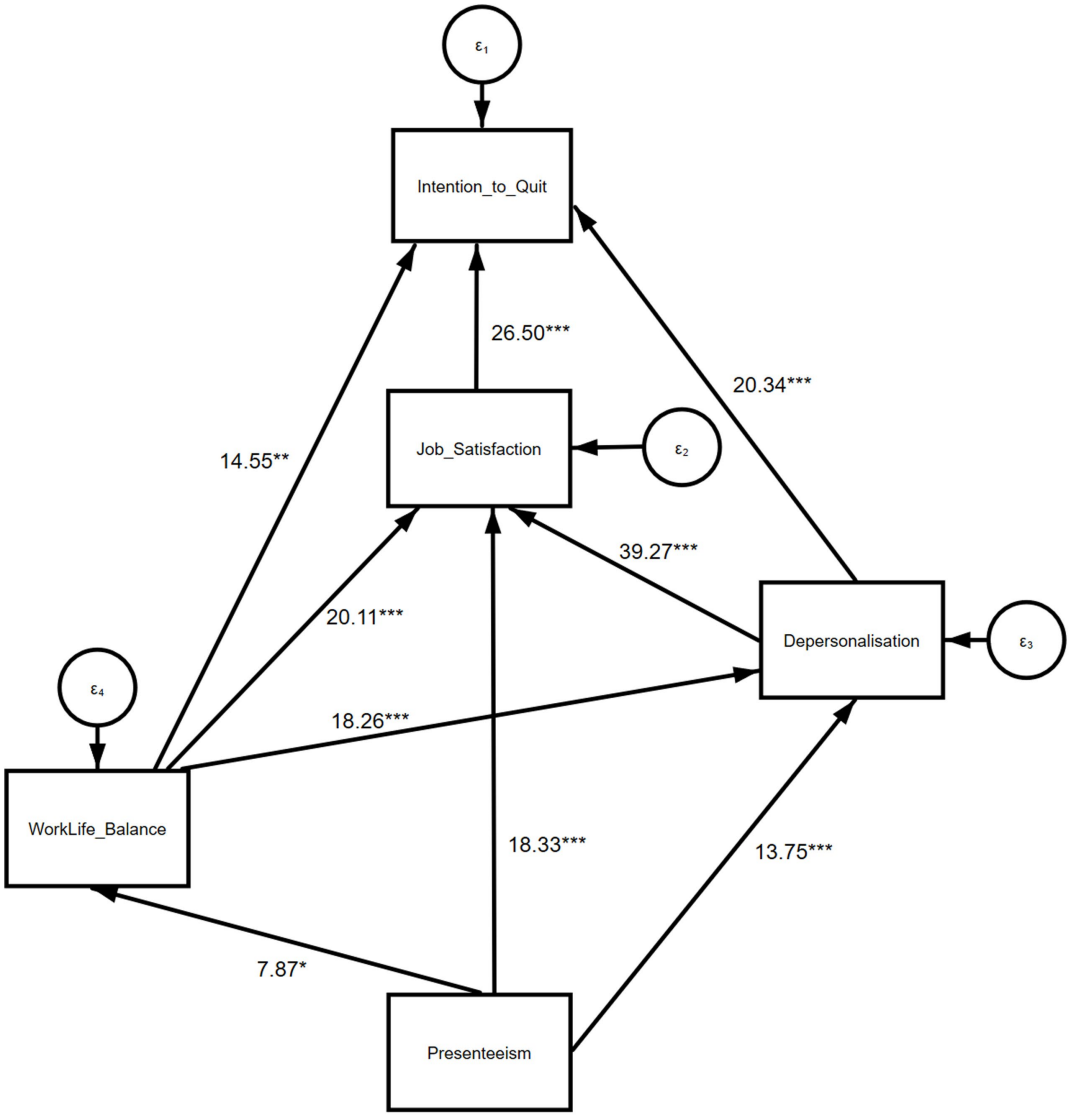
Structural model with depersonalisation frequency as the outcome^††^. ^††^*n* = 351; values represent the chi-squared test for statistical significance, where **p ≤ 0.001, **p < 0.01, *p < 0.05.

The GSEM routine employed in our analysis does not enable reporting the usual indices of model fit. Instead, we present the final adjusted GSEM results, including the values of the chi-squared test for statistical significance, in Figure 1 (emotional exhaustion and career disengagement factors) and 2 (depersonalisation and career disengagement factors), as well as in Tables 2, 3. Due to the ordinal nature of the response variables, we focused on *p*-values and statistical significance to assess the overall association between any pair of variables, rather than the standardized path coefficients. All direct paths in the model (Figures 1, 2) demonstrated a significant association, except for the one between work–life balance and presenteeism in the model for GPs aged 50 or younger. Therefore, we focused on the strongest associations for each outcome, corresponding to the variable immediately below the outcome in the ‘hierarchy of prediction’ (Tables 2, 3).

**Table 2:** Broad summary of the GSEM findings at the GP level: emotional exhaustion frequency.**

Covariate	Outcome			
All respondents (*N* = 351)	ITQ	Job satisfaction	Emotional exhaustion frequency	Work–life balance
Job satisfaction	**χ**^2^ **= 23.48** **d.f. = 3** **p < 0.001**			
Emotional exhaustion frequency	χ^2^ = 24.66 d.f. = 5 *p* < 0.001	**χ**^2^ **= 44.84** **d.f. = 5** **p < 0.001**		
Work–life balance	χ^2^ = 7.41 d.f. = 3 *p* = 0.060	χ^2^ = 11.74 d.f. = 3 *p* = 0.008	**χ**^2^ **= 46.76** **d.f. = 3** **p < 0.001**	
Presenteeism		χ^2^ = 15.46 d.f. = 2 *p* < 0.001	χ^2^ = 18.83 d.f. = 2 *p* < 0.001	**χ**^2^ **= 7.87** **d.f. = 2** **p = 0.020**
Respondents aged ≤ 50 years (N = 249)				
Job satisfaction	**χ**^2^ **= 28.04** **d.f. = 3** **p < 0.001**			
Emotional exhaustion frequency	χ^2^ = 15.55 d.f. = 5 *p* = 0.008	**χ**^2^ **= 34.66** **d.f. = 5** **p < 0.001**		
Work–life balance	χ^2^ = 7.38 d.f. = 3 *p* = 0.061	χ^2^ = 10.23 d.f. = 3 *p* = 0.017	**χ**^2^ **= 26.93** **d.f. = 3** **p < 0.001**	
Presenteeism		χ^2^ = 11.77 d.f. = 2 *p* = 0.003	χ^2^ = 11.24 d.f. = 2 *p* = 0.004	**χ**^2^ **= 1.51** **d.f. = 2** **p = 0.469**

** *p*-values were rounded to the third decimal. Values in bold correspond to the covariate with the strongest association with each outcome in the path diagram.

**Table 3:** Broad summary of the GSEM findings at the GP level: depersonalisation frequency.***

Covariate	Outcome			
All respondents (*N* = 351)	ITQ	Job satisfaction	Depersonalisation frequency	Work–life balance
Job satisfaction	**χ**^2^ **= 26.50** **d.f. = 3** **p < 0.001**			
Depersonalisation frequency	χ^2^ = 20.34 d.f. = 5 *p* = 0.001	**χ**^2^ **= 39.27** **d.f. = 5** **p < 0.001**		
Work–life balance	χ^2^ = 14.55 d.f. = 3 *p* = 0.002	χ^2^ = 20.11 d.f. = 3 *p* < 0.001	**χ**^2^ **= 18.26** **d.f. = 3** **p < 0.001**	
Presenteeism		χ^2^ = 18.33 d.f. = 2 *p* < 0.001	χ^2^ = 13.75 d.f. = 2 *p* = 0.001	**χ**^2^ **= 7.87** **d.f. = 2** **p = 0.020**
Respondents aged ≤ 50 years (N = 249)				
Job satisfaction	**χ**^2^ **= 33.72** **d.f. = 3** **p < 0.001**			
Depersonalisation frequency	χ^2^ = 13.24 d.f. = 5 *p* = 0.021	**χ**^2^ **= 25.56** **d.f. = 5** **p < 0.001**		
Work–life balance	χ^2^ = 10.19 d.f. = 3 *p* = 0.017	χ^2^ = 16.04 d.f. = 3 *p* = 0.001	**χ**^2^ **= 13.27** **d.f. = 3** **p = 0.004**	
Presenteeism		χ^2^ = 14.94 d.f. = 2 *p* < 0.001	χ^2^ = 7.90 d.f. = 2 *p* = 0.019	**χ**^2^ **= 1.51** **d.f. = 2** **p = 0.469**

***p-values were rounded to the third decimal. Values in bold correspond to the covariate with the strongest association with each outcome in the path diagram.

In the first model exploring the path relationship between emotional exhaustion and career disengagement factors (Figure 1), intention to quit patient care had the strongest association with job satisfaction (χ2 = 23.48, *p* < 0.001, df = 3), followed by emotional exhaustion (χ2 = 24.66, *p* < 0.001, df = 5). The association between the intention to quit patient care and work–life balance was weaker (χ2 = 7.41, *p* = 0.060, df = 3). Job satisfaction showed the strongest association with emotional exhaustion (χ2 = 44.84, *p* < 0.001, df = 5), but it was also significantly associated with work–life balance (χ2 = 11.74, *p* = 0.008, df = 3) and presenteeism (χ2 = 15.46, *p* < 0.001, df = 2). For emotional exhaustion, the strongest association was observed with work–life balance (χ2 = 46.76, *p* < 0.001, df = 3), followed by presenteeism (χ2 = 18.83, *p* < 0.001, df = 2), and for work–life balance, the strongest association was observed with presenteeism (χ2 = 23.48, *p* < 0.001, df = 2).

In the second model exploring the path relationship between depersonalisation and career disengagement factors (Figure 2), intention to quit patient care had the strongest association with job satisfaction (χ2 = 26.50, *p* < 0.001, df = 3), followed by depersonalisation (χ2 = 20.34, *p* = 0.001, df = 5) and work–life balance (χ2 = 14.55, *p* = 0.002, df = 3). We observed the strongest association between job satisfaction and depersonalisation (χ2 = 39.27, *p* < 0.001, df = 5). The associations between work–life balance (χ2 = 20.11, *p* < 0.001, df = 3) and presenteeism (χ2 = 18.33, *p* < 0.001, df = 2) were also strong, although comparatively weaker. For depersonalisation, the strongest association was observed with work–life balance (χ2 = 18.26, *p* < 0.001, df = 3), followed by presenteeism (χ2 = 13.75, *p* = 0.001, df = 2). Similar associations were found across the two models for the subgroup of GPs aged 50 or younger.

## Discussion

### Summary of the findings

Our study outlines a pathway that identifies a spectrum of associations, ranging from the most direct and immediate to the most indirect and distant, between burnout and career disengagement factors among GPs in England. We found that job satisfaction and the two dimensions of burnout (emotional exhaustion and depersonalisation) were the only direct and immediate factors associated with intention to quit patient care, which is considered the most proximal indicator of career disengagement. Other common career disengagement factors, such as work–life balance and presenteeism, were primarily associated with the intention to quit patient care indirectly through burnout. In addition, the demographic characteristics of GPs did not contribute to this pathway beyond career disengagement factors and burnout dimensions.

### Strengths and limitations of the study

Our study employed advanced statistical techniques to explore the complex associations between burnout dimensions and career disengagement factors among GPs in England. However, several limitations should be noted. First, the cross-sectional study design precludes the establishment of causation, emphasising the need for future studies employing longitudinal data. Second, the response rate across practices was low (39%), although the rate is higher than that in most studies involving GP respondents (28, 29) and substantially higher than that in the UK’s Tenth National GP Work-life Survey in 2019 (30). For example, a large cross-sectional survey of burnout among physicians in the US (31) reported a response rate of approximately 20%, whereas the GP Work-life Survey had a cross-sectional response rate of 12% in 2019. However, we cannot rule out the possibility that our findings reflect only the perspectives of the participating GPs, rather than those of all practising GPs across the UK. Third, although the survey used validated items, the potential risk of selection and/or recall bias cannot be ruled out, especially if responding GPs had already been experiencing emotional exhaustion or depersonalisation in the preceding 12 months. While the demographic characteristics of the respondents broadly align with national statistics for GPs, the voluntary nature of the survey might have introduced selection bias. Therefore, caution should be exercised when generalising the findings to the wider GP workforce in England. Furthermore, the survey was distributed during the very early stages of the COVID-19 pandemic, which might have introduced unique stressors that influenced the GPs’ responses, particularly regarding burnout, disengagement, and intention to quit patient care (32). However, as major disruptions had not yet fully materialised during most of the data collection period, the early pandemic context should be considered when interpreting the results. Fourth, the distribution of the questionnaires at the onset of the COVID-19 pandemic might have influenced the responses due to increased workload pressures. Fifth, we used a two-item abbreviated measure of burnout to shorten the survey and reduce the risk of dropout. This measure has demonstrated excellent value in capturing overall burnout as well as emotional exhaustion and depersonalization dimensions of burnout in physicians. However, this measure did not include the personal accomplishment dimension of the MBI, and there might be additional benefits to including this dimension, especially when examining paths to positive outcomes such as fulfilment and motivation. We recommend replicating these findings using the full MBI measure. Moreover, we acknowledge that practice-and country-level factors, such as healthcare policies and organisational culture and teamwork (33), were not accounted for and might have influenced levels of burnout and career disengagement. Future research should incorporate these potential confounders for a more comprehensive understanding of the tested relationships.

### Comparison with existing literature

Work stress and burnout in doctors are often associated with suboptimal patient safety (7, 14, 34) and career disengagement (14). However, little is known about the mechanisms underlying the association between burnout and career disengagement. Our findings support the Job Demands-Resources model by demonstrating that high levels of burnout (emotional exhaustion and depersonalisation) directly contribute to the intention to quit patient care, a critical indicator of career disengagement. The above model suggests that job demands, such as excessive workload and poor work–life balance, act as stressors that lead to burnout, which, in turn, exacerbates disengagement from patient care. Our study reinforces this finding by demonstrating that burnout is strongly associated with job dissatisfaction, which is the most direct factor linked to quitting intention.

In the UK, the primary care workforce has been severely affected by prolonged staff shortages, funding shortfalls, and poor planning (35). Recent studies have revealed an increasing number of doctors retiring or leaving direct patient care early in their careers (36). In addition, working during the COVID-19 pandemic has also increased burnout levels among GPs (37). Burnout and job satisfaction are known drivers of the intention to quit patient care among GPs (5, 15, 38). This study advances these findings by offering a potential pathway through which burnout dimensions and career disengagement factors intercorrelate among GPs. Our findings suggest that GP retention may be at immediate risk when burnout and job dissatisfaction are high, while poor work–life balance and presenteeism may serve as opportunities for early intervention to improve GP retention.

Job satisfaction is typically defined as an individual’s perceptions and evaluation of their job, and these perceptions are influenced by the demands, values and expectations associated with their job (15). We propose that GPs with higher job satisfaction are more enthusiastic about work and derive greater utility from role-related tasks, which, in turn, is likely to reduce their intention to quit. There is also evidence that burnout is driven by working conditions, such as excessive demands, toxic cultures, and poor working environments (18, 39, 40). GPs have one of the highest rates of poor work–life balance among medical professionals (41), likely influencing their decisions to reduce working hours and retire early in their careers (42, 43). Doctors may also have a higher threshold for recognising illness in themselves, often reserving sick leave for when their dependents are unwell (44). Building on these findings, we demonstrated that work–life balance and presenteeism have an indirect effect on the intention to quit patient care via emotional exhaustion and depersonalisation dimensions of burnout (45, 46). Some studies have suggested that perceived social support in the workplace is associated with lower levels of sickness presenteeism (47). It may be that interventions aimed at reshaping doctors’ attitudes toward work–life balance and sickness will have a positive impact on GP well-being and retention (48). Investing in such organisational changes is recommended by our findings.

### Implications for research and practice

Job dissatisfaction and burnout are likely immediate indicators that GPs are at high risk of quitting direct patient care. High levels of burnout and job dissatisfaction may require urgent action to improve GP retention. Strategies such as fair compensation, professional development, and a supportive work environment could enhance job satisfaction. In addition, providing sufficient organisational support, including time and resources to implement stress management programmes and mental health support, can potentially reduce burnout and help retain GPs.

Poor work–life balance and presenteeism may serve as early indicators of the intention to quit, as they are associated with burnout and job dissatisfaction. Preventative measures, such as offering flexible working hours and fostering a supportive culture that encourages taking sick leave without stigma, can serve as early interventions to improve work–life balance and reduce presenteeism.

Interestingly, demographic factors of GPs do not significantly contribute to the path toward career disengagement. Therefore, efforts should focus on addressing work stress and the workplace environment.

Extending this pathway to include work culture and practice-level characteristics, as well as adopting a longitudinal approach with a larger sample of GPs and practices, is recommended. This finding would provide insights into how burnout, work culture, and other practice-specific characteristics influence self-reported intentions to quit patient care and actual career disengagement in GPs. These insights would also enable causal mechanisms to be established and subsequently to be more targeted and comprehensive interventions to enhance GP retention.

## Data Availability

The datasets presented in this article are not readily available because this study used pseudonymised patient-level data from the Oxford-Royal College of General Practitioner Research and Surveillance Centre (RSC). These data can be accessed for ethically approved research by applying via https://orchid.phc.ox.ac.uk/.
