# Supplementary material for: Associations between burnout and career disengagement factors among general practitioners: a path analysis
Source: Front Public Health. 2025 Jun 18;13:1547102. doi: 10.3389/fpubh.2025.1547102 (PMC12213890; doi:10.3389/fpubh.2025.1547102)
Supplement: Supplementary file 1 [file Data_Sheet_1.docx]

**Supplementary file 1 -** **Associations between burnout and career disengagement factors in general practitioners: A path analysis.**

**Information S1 – GP Wellbeing Questionnaire**

1. **What is your age?**

|  |
| --- |

1. **What is your gender?**

|  |
| --- |

1. **What is your full-time (FTE%) equivalent ?**

|  |
| --- |

1. **What is the full-time equivalent (FTE%) of all GPs in your practice?**

|  |
| --- |

1. **How many years have you worked in your practice?**

|  |
| --- |

1. **How often you feel burned out from your work?^1^**

| Never | A few times a year or less | Once a month or less | A few times a month | Once a week | A few times a week | Every day |
| --- | --- | --- | --- | --- | --- | --- |

1. **How often you feel that you have become callous toward people since you took this job?^1^**

| Never | A few times a year or less | Once a month or less | A few times a month | Once a week | A few times a week | Every day |
| --- | --- | --- | --- | --- | --- | --- |

1. **Have you gone to work with an illness in a situation where you would have recommended a patient to stay home during the last 12 months?^2^**

| None | Once | Two to four  times | More than five times |
| --- | --- | --- | --- |

1. **Your work schedule leaves you enough time for your personal/family life^3^?**

| Strongly agree | Agree | Neutral | Disagree | Strongly disagree |
| --- | --- | --- | --- | --- |

1. **You are satisfied with your career in general practice?^4^**

| Strongly agree | Agree | Neutral | Disagree | Strongly disagree |
| --- | --- | --- | --- | --- |

1. **What is the likelihood you will leave direct patient care within five years?**

| None | Slight | Moderate | Considerable | High |
| --- | --- | --- | --- | --- |

1. West CP, Dyrbye LN, Sloan JA, Shanafelt TD. Single item measures of emotional exhaustion and depersonalization are useful for assessing burnout in medical professionals. *J Gen Intern Med*. 2009;24(12):1318-1321.

2. Thun S, Fridner A, Minucci D, Løvseth LT. Sickness present with signs of burnout: The relationship between burnout and sickness presenteeism among university hospital physicians in four European countries. *Scandinavian Psychologist*. 2014;1.

3. Shanafelt TD, Boone S, Tan L, et al. Burnout and Satisfaction With Work-Life Balance Among US Physicians Relative to the General US Population. *Arch Intern Med*. 2012;172(18):1377-1385.

4. Neumann JL, Mau L-W, Virani S, et al. Burnout, Moral Distress, Work–Life Balance, and Career Satisfaction among Hematopoietic Cell Transplantation Professionals. *Biology of Blood and Marrow Transplantation*. 2018;24(4):849-860.

**Table S1 – GP-Level Modelling (multi-level ordered logistic regression)**

| Covariate | Outcome Variable | | | |
| --- | --- | --- | --- | --- |
| P-values for GP Characteristics-only Models | ITQ | Emotional exhaustion Freq. | Job Satisfaction | Work-Life Balance |
| Age (Linear) | 0.047 (L) | 0.060 (Q) | 0.026 (Q) | 0.111 (Q) |
| Age (Quadratic) |  |  |  |  |
| Gender | 0.348 | 0.610 | 0.735 | 0.187 |
| FTE | 0.573 | 0.743 | 0.888 | 0.003 |
| Years at Practice | 0.609 | 0.728 | 0.429 | 0.996 |

**Correlations and clustering**

We observed a moderate association between the two dimensions of burnout, emotional exhaustion and depersonalisation (Tau=0.51; P < 0.001), as well as with job satisfaction (Tau=0.45; P < 0.001 and Tau=0.36; P < 0.001, respectively). Regarding both emotional exhaustion and depersonalisation, the weakest associations were with presenteeism (Tau=0.24; P < 0.001 and Tau=0.22; P < 0.001, respectively). Furthermore, we noted weak to moderate correlations between intention-to-quit and job satisfaction (Tau=0.39; P < 0.001), intention-to-quit and work-life balance (Tau=0.31; P < 0.001), and job satisfaction with work-life balance (Tau=0.33; P < 0.001). The weakest associations were observed between presenteeism and work-life balance (Tau=0.22; P < 0.001) and presenteeism and intention-to-quit (Tau=0.20; P < 0.001).

**Table S2 – Correlation between job satisfaction, emotional exhaustion freq., and turnover intention of the Primary Care Practitioners (PCPs)**

|  | Correlation – Kendall’s Tau-b | | | | |
| --- | --- | --- | --- | --- | --- |
| **All Respondents**  **(N = 351)** | ITQ | Job Satisfaction | Emotional exhaustion Freq. | Depersonalisation Freq. | Work-Life Balance |
| **Job satisfaction** | 0.3882 |  |  |  |  |
| **Emotional exhaustion freq.** | 0.3656 | 0.4485 |  |  |  |
| **Depersonalisation Freq.** | 0.2431 | 0.3594 | 0.5066 |  |  |
| **Work-life balance** | 0.3098 | 0.3308 | 0.3668 | 0.2397 |  |
| **Presenteeism** | 0.1981 | 0.2458 | 0.2438 | 0.2167 | 0.2171 |

**Table S3 – Intra-class correlations (within-practice clustering*)**

| Practice Size | Individual Responses | Practice Responses | Emotional exhaustion Freq. | ITQ | Job Satisfaction | Work-Life Balance |
| --- | --- | --- | --- | --- | --- | --- |
| All | 351 | 57 | .0893 | .0708 | .1214 | .1033 |
| ≥ 2 GPs | 337 | 49 | .0745 | .0633 | .1078 | .1072 |
| ≥ 3 GPs | 321 | 41 | .0842 | .0605 | .1028 | .1050 |
| ≥ 5 GPs | 268 | 36 | .0756 | .0000 | .0426 | .0901 |
| ≥ 7 GPs | 171 | 18 | .0141 | .0180 | .0000 | .0399 |

* Intra-Practice Correlation Coefficients calculated from a ‘Large’ One-Way ANOVA.
